# Supplementary material for: Prospective cohort study of surgical site infections following single dose antibiotic prophylaxis in caesarean section at a tertiary care teaching hospital in Medchal, India
Source: PLoS One. 2024 Jan 25;19(1):e0286165. doi: 10.1371/journal.pone.0286165 (PMC10810521; doi:10.1371/journal.pone.0286165)
Supplement: S1 Questionnaire — (PDF) [file pone.0286165.s001.pdf]

SHARE INDIA

MediCiti Institute of Medical Sciences Campus

Ghanpur,Medchal,R.R.Dist-501401 T.S

QUESTIONNAIRE

CSSI ID:Interviewer's Name / ID : ______________

Date of Abstracting : __ __/ __ __ /__ __ __ __Record the Time: ___ ___ / ___ ___
(dd/mm/yyyy) Hours Minutes

Q.NO.	QUESTION	RESPONSE
1.	**Name Surname**<br>**Given name**	
2.	**Wife of surname**<br>**given name**	
3.	**Age in years**	
4.	**Height in cm**	................cm<br>☐ 999.not recorded
5.	**Weight in kg**	_ _ _ . _ _kg<br>☐ 999.not recorded
6.	**Contact no.**	1.Maternal contact no.-<br>2.Husband contact no.-
7.	**Address**	
8.	**IP Number**	
9.	**Unit**	☐ 1.I<br>☐ 2.II
10.	**Diagnosis**	
11.	**Place of admission**	☐ 1. General Ward<br>☐ 2. Special Room<br>☐ 3.Labour room
12.a	**Booking status**	☐ 1. Booked<br>☐ 2. Unbooked
12.b	**If booked case no. Of prenatal visits**	____
13.	**Date of admission in Ward**	__ __/ __ __/__ __ __ __
		dd / mm / yyyy

14.	**Date of discharge**	__ __ /__ __ / __ __ __ __<br><br>dd / mm / yyyy
15	**Duration of hospital stay**	days
16.	**HB in gm/dl**	Value..............<br>Date................
17.	**Blood glucose in mg/dl**<br>☐ **FBS**<br>☐ **RBS**	Value …………..<br>Date---------------
18.	**Co-morbid Conditions**	(see table below)

S.no.	Co-morbid Condition	0 (No)	1 (Yes)	
A1	Pre existingDiabetes			
A2	Gestational diabetes			
B	Hypertension			
C	Hypothyroidism			
D	Asthma			
E	COPD			
F	Chronic kidney disease			
G	HIV			
H	STD			
I	Others-spfy			
J	None			

19.	**Risk factors**	☐ A.Anemia (anemia)<br>☐ B.PROM (prom)<br>☐ C.Prolonged pre-op stay (pre-op stay)<br>☐ D.Prolonged stay after surgery (post-op stay)<br>☐ E.Multiple vaginal examination (mve), number<br>☐ F.Gestational diabetes (gd)<br>☐ G.Meconium stained liquor<br>☐ H.Others ----spy----------------<br>☐ I.None
20.a	**Antibiotic given in labour**	☐ 1.yes<br>☐ 2.no
20.b	**if yes, indication for giving antibiotic**	
20.c	**which antibiotic**	

20.d	**Number of doses given**	☐ 1<br>☐ 2<br>☐ 3<br>☐ 4<br>☐ More Specify-----------
21.a	**Antenatal steroid given**	☐ 1.Yes ☐ 2.No
21.b	**If steroid given the time interval between administration and surgery**	_____
22.	**Duration of labor**	☐ 1.<6H<br>☐ 2.6-12H<br>☐ 3.>12H<br>☐ 4.>18H<br>☐ 5.>24h<br>☐ **6.** No labor<br>☐ **999.** Not recorded
23.	**Duration of rupture of membranes in hrs**	☐ 1.Elective ROM<br>☐ 2.<6 hrs<br>☐ 3.6-12 rs<br>☐ 4.12-18 hrs<br>☐ 5. 18-24 hrs<br>☐ 6.>24 hrs<br>☐ 999. Not recorded
24.a	**Clinical diagnosis of chorioamniotis in labour**	☐ 1.Yes ☐ 2.No

24.b	**Diagnostic criteria of chorioamniotis (atleast 2)**	☐ 1.Temp.>38°C<br>☐ 2.Maternal tachycardia(>120BPM)<br>☐ 3.fetal tachycardia(160-180BPM)<br>☐ 4.uterine tenderness<br>☐ 5. foul smelling discharge<br>☐ 6. leucocytosis. WBC>15,000-18,000 cells/µL
25.	**Previous Surgeries(names and no.)**	1.Ceasarean __ **0** __ **1** __ **2** __ **3+**<br>2.Laparotomy __ **0** __ **1** __ **2**<br>☐ 999.not recorded
26.	**Hair removal**	☐ 1. Shaving:<br>☐ 2.Trimming / Clipping :<br>☐ 3.None<br>☐ 999.Not recorded
27.	**If hair removed, how many hrs before surgery?**	------------hrs ☐ 999.Not recorded
28.	**Date of surgery**	__ __ / __ __ / __ __ __ __
29.	**Start time (Time of Skin Incision)**	__ __ : __ __<br>☐ 00 : 00
30.	**End Time of surgery**	__ __ : __ __<br>☐ 00:00
31	**Duration of surgery**	__ __ : __ __
32.	**Type of skin disinfectant used**	☐ 1. Povidine Iodine<br>☐ 2. Chlorhexidine with alcohol<br>☐ 3. 70% alcohol<br>☐ Others...spy------------
33.	**RBS at the time of surgery**	----------mg/dl<br>☐ 999.Not recorded

34.	**Type of anesthesia**	☐ 1. Spinal anesthesia<br>☐ 2. Epidural anesthesia<br>☐ 3. General anesthesia
35.	**ASA score**	☐ 1<br>☐ 2<br>☐ 3<br>☐ 4<br>☐ 5
36.	**Type of LSCS**	☐ 1. Emergency<br>☐ 2. Elective
37.	**Type of incision**	☐ 1. SUMI<br>☐ 2. Pfannensteil
38.	**Indication of LSCS**	☐ A. Previous LSCS with CPD<br>☐ B. Malpresentation<br>☐ C. Fetal distress<br>☐ D. Oligohydramnios with IUGR<br>☐ E. Precious pregnancy<br>☐ F. CPD<br>☐ G. Non progress of labour<br>☐ H. Deep transverse arrest<br>☐ I. Failed induction of labour<br>☐ J. Placenta previa<br>☐ K. Abruptio placenta<br>☐ L. Others----spy--------<br>☐ M. None of the above

39a.	**Operating surgeon**	☐ PG<br>☐ Asst.Prof.<br>☐ Assoc.Prof<br>☐ Prof.
39 b.	**Assisting surgeon**	☐ PG<br>☐ Asst. Prof<br>☐ Assoc. Prof.<br>☐ Prof.
40	**Number of people in OT**	☐ 5<br>☐ 10<br>☐ 15<br>☐ >15
41	**Exteriorisation of uterus**	☐ 1.Yes<br>☐ 2. No
42	**Delivery by forceps**	☐ 1.Yes<br>☐ 2. No
43	**Use of cautery in sub cutaneous tissue**	☐ 1.Yes<br>☐ 2.No
44	**Placental removal**	☐ Spontaneous<br>☐ Manual
45.a	**Type of skin closure (select one only)**	☐ 1. Sub cuticular<br>☐ 2. Mattress
45.b	**Suture material used for skin closure (select all)**	☐ 1. Sutupack<br>☐ 2. Silk<br>☐ 0. Monocryl<br>☐ Others---spy------------------

46.a	**Was a prophylactic antibiotic administered?**	☐ 1. yes ☐ 2. no
46.b	**b. Time of administration of prophylactic antibiotic**	__ __ : __ __<br>☐ 00:00 (not recorded)
46.c	**Duration of antibiotic prior to skin incision**	__ __ : ___ ___
47.	**Antibiotic prophylaxis (Drug Name and Dosing)**	☐ 1. Cefazolin 1g iv single dose<br>☐ Others---specify--------------------
48.	**Intra operative blood Transfusion**	☐ 1.yes ☐ 2. no
49.	**Type of wound**	☐ 1.Class1(clean)<br>☐ 2.Class 2 (clean contaminated)<br>☐ 3.Class 3 (contaminated)<br>☐ 4.Class4 (dirty)
50. a	**Post operative dressing done**	☐ 1.yes ☐ 2. no
50. b	**If yes, date of dressing**	__ __ / __ __ / __ __ __ __
50. c	**Type of disinfectant used for dressing**	☐ 1. Betadine<br>☐ 2. Spirit
51.a	**SSI present**	☐ 1.yes ☐ 2.no
51.b	**Type of SSI, If present**	☐ 1. Superficial ☐ 3. Organ space<br>☐ 2.Deep
51.c	**Post operative day of diagnosis of SSI**	
52.a	**UTI present**	☐ 1.yes ☐ 2. no
52.b	**UTI onset day**	
53.a	**Sepsis seen**	☐ 1.yes ☐ 2.no
53.b	**Sepsis diagnostic criteria(atleast 2)**	☐ 1.hyper >38.3°C/hypothermia<36°C<br>☐ 2.tachycardia>90bpm<br>☐ 3.leucocytosis(>12,000 µL-1)/ Leukopenia (<4000µL)<br>☐ 4.Acutely Altered Mental Status<br>☐ 5.Tachypnea >20 bpm<br>☐ 6.Hyperglycemia (>120 mg/dl) in the absence of diabetes

54.	**Type of specimen collected**	☐ 1.pus<br>☐ 2.urine<br>☐ 3.blood
55.a	**Bacterial culture isolate from pus**	☐ A. Staphylococcus aureus<br>☐ B. CONS<br>☐ C. Enterococcus sps.<br>☐ D. Streptococcus sps.<br>☐ E. Escherichia coli<br>☐ F. Klebsiellasps<br>☐ G. Enterobactersps<br>☐ H. Proteus sps.<br>☐ I.Pseudomonassps<br>☐ J.Acinetobactersps<br>☐ K.Others...spy----<br>☐ L.None
55.b	**Bacterial culture isolate from urine**	☐ A. Staphylococcus aureus<br>☐ B. CONS<br>☐ C. Enterococcus sps.<br>☐ D. Streptococcus sps.<br>☐ E. Escherichia coli<br>☐ F. Klebsiellasps<br>☐ G. Enterobactersps<br>☐ H. Proteus sps.<br>☐ I.Pseudomonassps<br>☐ J.Acinetobactersps<br>☐ K.Others...spy----<br>☐ L.None

55.c	**Bacterial culture isolate from blood**	☐ A. Staphylococcus aureus<br>☐ B. CONS<br>☐ C. Enterococcus sps.<br>☐ D. Streptococcus sps.<br>☐ E. Escherichia coli<br>☐ F. Klebsiella spp<br>☐ G. Enterobacter spp<br>☐ H. Proteus spp.<br>☐ I.Pseudomonas spp<br>☐ J.Acinetobacter spp<br>☐ K.Others...spy----<br>☐ L. None
56.	**Antibiogram of gram positive bacteria-1**	(see table below)
57.	**Antibiogram of gram negative bacteria-2**	(see table below)

56. Antibiogram of gram positive bacteria-1

S.no.	Antibiotic	Sensitive	Resistant	
1.	Penicillin			
2.	Ampicillin			
3.	Amoxy-clav			
4.	Erythromycin			
5.	Clindamycin			
6.	Co-trimoxazole			
7.	Cephalexin			
8.	Cefoxitin			
9.	Vancomycin			
10.	Gentamicin			
11.	Linezolid			
12.	Ampicillin+ sulbactam			

57. Antibiogram of gram negative bacteria-2

S.no.	Antibiotic	Sensitive	Resistant	
1.	Ampicillin			
2.	Ciprofloxacin			
3.	Co-trimoxazole			
4.	Amikacin			
5.	Ceftriaxone			
6.	Ceftazidime			
7.	Ceftazidime+ clavulanic			
8.	Cefoxitin			
9.	Piperacillin+ tazobactam			
10.	Imipenem			

	**Antibiogram– 3**	S.no.	Antibiotic	Sensitive	Resistant
		1.	Penicillin		
		2.	Ampicillin		
		3.	Amoxy-clav		
		4.	Erythromycin		
		5.	Clindamycin		
		6.	Co-trimoxazole		
		7.	Cephalexin		
		8.	Cefoxitin		
		9.	Vancomycin		
		10.	Gentamicin		
		11.	Tetracycline		
		12.	Ampicillin+sulbac tam		
	**Antibiogram-4**	S.no.	Antibiotic	Sensitive	Resistant
		1.	Ampicillin		
		2.	Ciprofloxacin		
		3.	Co-trimoxazole		
		4.	Amikacin		
		5.	Ceftriaxone		
		6.	Ceftazidime		
		7.	Ceftazidime+ clavulanic		
		8.	Cefoxitin		
		9.	Piperacillin+ tazobactam		
		10.	Imipenem		

58. Treatment:

S.no.	Tick the chosen Antibiotic	Drug name	Dose	Start date	End date	Total duration
A.		Ampicillin				
B		Ampicillin				
C		Metronidazole				
D		Augmentin				
E		Cefotaxime				
F		Cefotaxime				
G.		Others---specify				
H		None				

59.	Resuturing done	☐ 1.Yes ☐ 2.No
