# Supplementary material for: Prospective cohort study of surgical site infections following single dose antibiotic prophylaxis in caesarean section at a tertiary care teaching hospital in Medchal, India
Source: PLoS One. 2024 Jan 25;19(1):e0286165. doi: 10.1371/journal.pone.0286165 (PMC10810521; doi:10.1371/journal.pone.0286165)
Supplement: Attachment — Submitted filename: Manuscript Revised.docx [file pone.0286165.s003.docx]

Prospective Cohort Study of Surgical Site Infections Following Single Dose Antibiotic Prophylaxis in Caesarean Section at a Tertiary Care Teaching Hospital in Medchal, India

Kalpana Basany^1^ *, Sirshendu Chaudhuri^2^, Lakshmi Shailaja^1^,^,≠a^,Varun Agiwal^2^, Neelima Angaali^1,≠b^, Nirupama AY ^2^Shailendra, D^1^, Catherine Haggerty^3^, PS Reddy^1,3^

^1^Department of Obstetrics and Gynecology, Society for Health Allied Research and Education, INDIA MediCiti Institute of Medical Sciences, Hyderabad, Telangana, India

^2^Department of Epidemiology, Indian Institute of Public Health, Hyderabad, Telangana, India

^1^Department of Microbiology, SHARE INDIA, MediCiti Institute of Medical Sciences, Hyderabad, Telangana, India Telangana, India

^1^Department of Pharmacology, SHARE INDIA, MediCiti Institute of Medical Sciences, Hyderabad, Telangana, India Telangana, India

^3^ Department of Epidemiology, University of Pittsburgh, Pittsburgh, PA

^3^ Division of Cardiology, Department of Medicine, University of Pittsburgh School of Medicine, University of Pittsburgh, Pittsburgh, PA

^≠a^Department of Obstetrics and Gynecology, Fernandez Hospital, Hyderabad, Telangana, India

^≠b^Department of Microbiology, Nizams Institute of Medical Sciences, Telangana, India

Corresponding Author: Kalpana Basany (KB)

[kalpanabasany@gmail.com](mailto:kalpanabasany@gmail.com)

**Abstract**:

Background: Caesarean section (CS) is a considered to be a life-saving operative intervention for women and new-borns in certain antepartum and intrapartum conditions. Caesarean delivery may be accompanied by several complications including surgical site infections (SSI). However, there is a significant lack of uniformity in the administration of antibiotics for preventing surgical site infections (SSI) following Caesarean deliveries. The present study was conducted to determine the incidence of post CS SSI following the adoption of single-dose antibiotic prophylaxis as recommended by WHO at a tertiary care teaching hospital in Medchal, India. Also, to identify the risk factors of SSI and reported the bacteriological profiles and the antimicrobial sensitivity and resistance pattern of the culture positive isolates

**Main objectives**: To estimate the incidence of surgical site infections (SSIs) according to CDC criteria following WHO-recommended single-dose antibiotic prophylaxis for Caesarean section at a tertiary care teaching hospital in Medchal, India.

**Methods**: A prospective hospital-based study was conducted between June 2017 and December 2019, in which women who underwent Caesarean delivery were followed up for 30 days post-delivery. Clinical details were collected using a structured questionnaire, and participants were followed up weekly after discharge to document any signs and symptoms of SSI. Symptomatic patients were requested to come to the hospital for further investigation and treatment. Standard microbiological tests were conducted to detect microorganisms and their antibiotic sensitivity.

**Results**: The study included 2,015 participants with a mean age of 24.1 years. The majority were multigravida (n=1,274, 63.2%) and underwent emergency Caesarean delivery (n=1,226, 60.8%). Of these, 92 participants (4.6%, 95% CI: 3.7% to 5.6%) developed surgical site infections, with 91 (98.9%) having superficial and 1 (1.1%) having a deep infection. Among those who developed an SSI, 84 (91.3%) did so during their hospital stay, while 8 (8.7%) developed an SSI at home. The adjusted relative risk (aRR) for developing an SSI was 2.5 (95% CI: 1.4 to 4.6; Power 99.9%) among obese women and 2.3 (95% CI: 1.1 to 4.7; Power 100%) among women aged 25 years or younger. Microbial growth was observed in 75.8% (n=50/66) samples. The most common organisms identified were *Staphylococcus aureus* (n=23, 46.0%), *Klebsiella sp*. (n=13, 26.0%), and *Escherichia coli* (n=12, 24.0%).

**Conclusion**: Given the low rate of SSI following Caesarean deliveries subjected to single-dose antibiotic prophylaxis and the increased risk noted with obesity, it is rationale to practice the latest recommendations of WHO including higher dose for obese patients, unless there is compelling evidence to do otherwise in any context.

**Manuscript**

Prospective Cohort Study of Surgical Site Infections Following Single Dose Antibiotic Prophylaxis in Caesarean Section at a Tertiary Care Teaching Hospital in Medchal, India

**Introduction**

Caesarean section (CS) is a considered to be a life-saving operative intervention for women and new-borns in certain antepartum conditions. Globally, CS is the most common major surgical intervention in pregnancies. [1] As per the World Health Organization (WHO), CS rate between 10-15% is considered optimum at the population level. Globally, the reported rate of CS is 21.1% and varies between 4.1% in West and Central Africa and 44.3% in the Latin America and the Caribbean region and increasing by 4% annually. [2,3] In India, the overall CS rate has increased from 8.5% to 21.5% in the last 15 years. [4] According to the last national-level survey, a substantial variation exists between the states- from as low as 5.2% in Nagaland to as high as 60.7% in Telangana. [4]

As a surgical procedure, Caesarean delivery may be accompanied by several complications including surgical site infections (SSI). [5] SSIs increase the morbidity and mortality of the mothers and babies and increases the length of hospital stay and thereby the cost of care. [6,7] To prevent SSI, the WHO recommend using a single dose antibiotic, mostly the first generation cephalosporins, before 30 to minutes of incision for all women undergoing CS. [8]

According to reports, the incidence of post-CS SSI worldwide is between 0.63% and 9.85%. [9-13] whereas in India, it ranges from 3.1% to 24.2%. [7,14–16] However, there is a significant lack of uniformity in the administration of antibiotics for preventing surgical site infections (SSI) following Caesarean deliveries.

Therefore, the present study was conducted to determine the incidence of post CS SSI following the adoption of single-dose antibiotic prophylaxis as recommended by WHO [8] in a tertiary care teaching hospital in Medchal, Telangana state, India. Prior to 2017, the hospital's practice involved administering post-operative antibiotics to all patients undergoing a Cesarean section (CS). However, after adapting the single-dose antibiotic prophylaxis, the administration of post-operative antibiotics was limited to cases where there was clinical or microbiological evidence of infection, specifically in cases of surgical site infections (SSIs). Additionally, we looked at the risk factors of SSI and reported the bacteriological profiles and the antimicrobial sensitivity and resistance pattern of the culture positive isolates.

**Methods**

**Setting:** The study was carried out at MediCiti Institute of Medical Sciences which is a tertiary care teaching hospital at Ghanpur village, located about 25km from the city of Hyderabad, in the Indian state of Telangana. The hospital has 570 inpatient beds out of which 60 were allocated to department of Obstetrics and Gynaecology. The caesarean section rate was 46% during the study period.

**Study design:** Prospective study

**Study duration**: The study was conducted between June 2017 & December 2019.

**Study population**: All women who underwent CS in the hospital during the study period were eligible for the study. We classified CS into two types- emergency and elective. An emergency CS is where there is either maternal or foetal compromise or where there is immediate threat to the life of women or foetus. Rest of the Caesarean deliveries were grouped under Elective CS. We excluded those women who delivered at other hospitals and came to the study hospital with surgical infection, those who died during CS or immediately after CS, and those who did not consent to participate.

**Sample size**: For estimating 2% or less incidence of SSI after CS, we estimated our minimum required sample size to be 1,500 women who undergo CS; considering α=0.05, β=0.8, & margin of error=0.01.

**Antibiotic policy of the hospital:** A single dose of prophylactic antibiotic was administered 30 min to one hour before the caesarean section. As a prophylaxis, we used Injection Cefazolin 1 gram. When Cefazolin was unavailable, injection Ampicillin 1 gram, OR injection Cefotaxim 1 gram was given. Post-operative antibiotics were not prescribed unless there was any clinical or bacteriological evidence of infection.

**Definition of variables**: SSI was defined as- "An infection of the superficial or deep skin incision, or of an organ or space, occurring up to 30 days after surgery.” [17] There are two types of SSI- superficial and deep. As per the CDC definition, "A superficial SSI is infection identified during hospital stay or within 30 days following caesarean section by readmission to the hospital or by post discharge survey in presence of any one of the following: purulent drainage with or without laboratory confirmation from the superficial incision; OR organisms isolated from an aseptically obtained culture of fluid or tissue from the incision; OR at least one of the following signs or symptoms: pain/ tenderness, localized swelling, redness, fever, redness, or heat and superficial incision is deliberately opened by surgeon, unless incision is culture-negative; OR Diagnosis of superficial incisional SSI made by a surgeon or attending physician.”

Deep surgical site infection is defined by CDC as- "Infection that occurs within 30 days after the operation if no implant is left in place or within one year if implant is in place and the infection appears to be related to the operation and infection involves deep soft tissue (e.g. fascia, muscle) of the incision and at least one of the following: Purulent drainage from the deep incision but not from the organ/space component of the surgical site; OR A deep incision spontaneously dehisces or is deliberately opened by a surgeon when the patient has at least one of the following signs or symptoms: fever (>38°C), localized pain or tenderness, unless incision is culture-negative; OR An abscess or other evidence of infection involving the deep incision is found on direct examination, during reoperation, or by histopathologic or radiologic examination; OR Diagnosis of deep incisional SSI made by a surgeon or attending physician.” All the suspected infections were confirmed either clinically by the treating gynaecologist or by the microbiologically by the culture results.

Body mass index (BMI) was calculated by weight in Kg divided by height in meter^2^. If height and weight measures were missed in the first trimester, or the participants recruited in the second trimester or later, BMI was excluded in the analysis. BMI was categorized according into Underweight (BMI < 18.5 kg/m^2^), Normal 1(BMI 18.5 to 22.93 kg/m^2^), Overweight (BMI 23 to 24.9 kg/m^2^, and Obese ≥25 kg/m^2^). (18) After admission, all women were assessed for co-morbid medical conditions like diabetes, hypertension, and thyroid disorders.

**Data collection**: All pertinent clinical details were collected by from the participants hospital record using structured questionnaire by a dedicated study nurse.

The hospital policy was to discharge the patient after suture removal on 6^th^ or 7^th^ postoperative day. The duration of hospital stay is from the day of admission to hospital to till the day of discharge from hospital. At the time of discharge from the hospital, the women were educated about the signs and symptoms of wound infection and were asked to report to the study nurse on noticing any signs and symptoms. The participants’ contact number(s) were collected before discharge. Post-discharge, the information on signs and symptoms were also collected by weekly telephone calls till the end of 30^th^ days from the date of operation by the study nurse. A standard script and a questionnaire in local language was used to enquire about the general health and wound infection. If suspected to have SSI, the women were followed up at the outpatient department (OPD).

In women where there was pus or discharge from the wound, two swabs were taken. Direct microscopy was done using the first swab, a smear was made on a clean glass slide and stained by Gram's stain. The second swab was inoculated onto plates of 5% sheep blood agar & Mac Conkey agar by rolling the swab over the agar and streaking from the primary inoculums, using a sterile bacteriological loop. If growth was seen after 24hrs, standard biochemical reactions were performed to isolate the organism. Antibiotic susceptibility testing (Antibiogram) of above isolates was performed by Kirby-Bauer disc diffusion method, using Muller Hinton agar plates according to Central Laboratory Standards Institute guidelines (CLSI). The same microbiological procedure was followed for those who were discharged and followed up in the OPD.

**Statistical analysis**: The data were analyzed by STATA version 14.0. Descriptive statistics in terms of mean and standard deviation (SD) or median and interquartile range (IQR) were used to characterize the participants. Incidence of SSI was calculated in terms of percentage with 95% confidence interval (CI). The determinants were assessed by univariate analysis followed by multiple logistic regression methods. Predictors with p-value <0.2 were considered for the final regression model. Risk of the predictors was estimated by adjusted relative risk (aRR) with 95% CI. A p-value <0.05 was considered significant for all statistical tests. Descriptive statistics were used to report the bacteriological profile of the SSI. Authors have access to deidentified data.

**Human subject protection:** The study was approved by the ethics committee of the institute. Besides, written informed consent was taken from all the participants.

**Results**

We recruited a total number of 2,038 participants in the study. We excluded 23 participants due to missing information. Finally, 2,015 participants’ information were available for analysis. The mean age of the participants was 24.1 years (SD 3.2 years), and majority were multigravida (n=1,274, 63.2%). (Table 1) Seven hundred eleven (35.3%) participants had at least one known Comorbidity Twelve hundred and twenty-six (60.8%) participants underwent emergency CS. The average hospital stay was 9.4 days (SD 3.2 days).

Table 1: Clinical profile of the participants, Medchal, Telangana, India (n=2,015)

| Variables | Frequency (%) |
| --- | --- |
| Age, years |  |
| <=25 | 1,415 (70.2) |
| >25 | 600 (29.8) |
| BMI*, Kg/m^2^ (n=1,356) |  |
| Underweight | 227 (16.7) |
| Normal | 604 (44.5) |
| Overweight | 221 (16.3) |
| Obesity | 304 (22.4) |
| Current pregnancy registered | 1803 (89.5) |
| Number of prenatal visits to hospital≥ 4 times | 1613 (80.1) |
| Presence of anaemia | 181 (9.0) |
| Presence of hypertension | 301 (14.9) |
| Presence of hypothyroidism | 268 (13.3) |
| Presence of gestational diabetes | 81 (4.0) |
| Previous Caesarean section | 1,124 (55.8) |
| Duration of labour (Hours) (n=2,010) |  |
| Non-labour | 849 (42.2) |
| <6 | 642 (31.9) |
| 6-12 | 381 (19.0) |
| >12 | 138 (6.9) |

*First trimester BMI is unavailable for 659 participants

***Incidence of SSI***:

Surgical site infection was developed in 92 (4.6%, 95% CI: 3.7% to 5.6%) out of 2015 participants. Out of this 91 (98.9%) had superficial and 1 (1.1%) had deep infection. Eighty-four (91.3%) participants developed infection during the hospital stay and eight (8.7%) at home. The infection rate was higher among women underwent emergency CS (n=70, 5.7%; 95% CI: 4.5 to 7.2) compared to those who underwent elective CS (n=22, 2.8%; 95% CI: 1.8 to 4.2).

The median time to develop SSI was 7 days (IQR 6 to 7 days). The average hospital stay was 11.8 days (SD 3.7 days) in women who developed SSI and 9.3 (SD 3.1) days without SSI and mean difference was 2.5 days (95% CI: 1.9 to 3.2 days; p <0.001).

***Risk factors***

Adjusted relative risk (aRR) of SSI was 2.3 times higher (95% CI: 1.1 to 4.7; Power 100%) among young age (<=25 years) and 2.5 times higher (95% CI: 1.4 to 4.6; Power 99.9%) among obese women. Women who underwent emergency LSCS (aRR 2.7; 95% CI: 1.0 to 7.6, p=0.06) had a higher SSI rate, though statistically not significant. (Table 2)

Table 2: Associated socio-demographic and clinical factors for developing the SSI

| Variable | Reference group | Study group | Univariate Logistic Regression | Multivariate Logistic Regression |
| --- | --- | --- | --- | --- |
|  |  |  | RR (95% CI) | aRR (95% CI) |
| Age (years) | >25 | <=25 | 1.8 (1.1-3.0) | 2.3 (1.1-4.7)* |
| BMI | Normal | Underweight | 0.7 (0.3-1.8) | 0.7 (0.3-1.7) |
|  |  | Overweight | 1.4 (0.7-2.9) | 1.3 (0.6-2.8) |
|  |  | Obesity | 2.4 (1.3-4.3) | 2.5 (1.4-4.6)* |
| Parity | Primi | Multi | 0.5 (0.3-0.7) | 0.6 (0.3-1.2) |
| Duration of labor (hours) | Non-labor | <6 | 1.5 (0.9-2.6) | 0.5 (0.2-1.3) |
|  |  | 6-12 | 2.1 (1.2-3.8) | 0.7 (0.2-1.8) |
|  |  | >12 | 3.3 (1.7-6.6) | 0.9 (0.3-2.8) |
| Type of LSCS | Elective | Emergency | 2.1 (1.3-3.4) | 2.7 (1.0-7.6) |
| No. of persons in OT | <5 | 5-10 | 0.3 (0.1-0.7) | 0.4 (0.1-1.1) |
|  |  | 11-15 | 0.3 (0.1-0.6) | 0.4 (0.1-1.3) |
|  |  | >15 | 0.2 (0.02-1.4) | 0.4 (0.04-4.0) |

***Bacteriological profile***

A total number of 66 wound discharge samples were sent for bacteriological assessment, out of which growth yielded in 50 (75.8%) samples- seven samples (14.0%) grew multiple organisms. We found *Staphylococcus aureus* (n=23, 46.0%)*, Klebsiella sp.* (n=13, 26.0%), and *Escherichia coli* (n=12, 24.0%) being the most common organisms. (Table 3)

The only gram-positive organism, Staphylococcus, was commonly sensitive to- Linezolid (20 isolates, 87%), Clindamycin (16 isolates, 69.6%), Levofloxacin (15 isolates, 65.2%), and Cefuroxime (14 isolates, 60.9%). (Table 3) Staphylococcus was commonly resistant to Penicillin (17 isolates, 73.9%), and Cotrimoxazole (10 isolates, 43.5%). (Table 3, 4) Among the gram-negative organisms, Klebsiella was the commonest organism. It was sensitive to Ceftazidime and clavulanic acid combination (13 isolates, 100%), Imipenem (10 isolates, 76.9%), and Cefepime (9 isolates, 69.2%) and commonly resistant to Ampicillin (11 isolates, 84.6%) and Amoxycillin/Clavulanic acid combination (10 isolates, 76.9%). (Table 3, 4)

**Table 3: Antimicrobial sensitivity pattern of the SSI isolates**

|  | *Staphylococcus (n=23)* | *Klebsiella (n=13)* | E.Coli (n=12) | *Citobacter (n=4)* | *Acinatobacter (n=3)* | *Proteus mirabilis (n=1)* | *Providencia (n=1)* |
| --- | --- | --- | --- | --- | --- | --- | --- |
| Amikacin |  |  |  |  |  |  | 1 |
| Amoxyclav | 7 | 3 | 1 | 3 |  |  | 1 |
| Amikacin |  | 5 | 4 | 3 | 2 |  |  |
| Ampicillin | 4 |  |  |  |  |  |  |
| Ampicillin/Sulbactum | 8 |  |  |  |  |  |  |
| Azithromycin | 6 |  |  |  |  |  |  |
| Aztreonam |  | 5 | 2 | 2 | 1 |  | 1 |
| Cefazolin | 2 |  |  |  |  |  |  |
| Cefepime | 1 | 9 |  | 2 | 1 |  | 1 |
| Cefixime |  | 1 |  |  |  |  | 1 |
| Cefoprerazone/Sulbactum |  |  | 1 | 1 |  |  |  |
| Cefotaxime | 1 | 5 |  |  |  |  | 1 |
| Cefoxitin | 10 | 4 | 4 |  |  |  | 1 |
| Ceftazidime/Clavulanic acid | 1 | 13 | 5 | 2 | 2 | 1 | 1 |
| Ceftriaxone |  | 4 |  | 1 | 2 |  |  |
| Cefuroxime | 14 |  |  |  |  |  |  |
| Ciprofloxacin | 11 | 8 | 1 | 2 | 1 | 1 | 1 |
| Clindamycin | 16 |  |  |  |  |  |  |
| Colistin |  | 1 |  |  | 1 |  |  |
| Co-Trimoxazole | 6 | 8 | 3 | 4 | 2 |  |  |
| Doxycycline | 9 | 2 | 1 | 2 | 1 |  | 1 |
| Erythromysin | 7 |  |  |  |  |  |  |
| Gentamycin |  | 3 | 1 | 1 |  |  |  |
| Imipenem | 1 | 10 | 10 | 3 |  | 1 | 1 |
| Levofloxacin | 15 |  |  |  |  |  |  |
| Linezolid | 20 |  |  |  |  |  |  |
| Meropenem |  | 3 | 1 | 1 | 4 |  |  |
| Netilmycin |  | 1 |  |  | 1 |  |  |
| Nitrofurantoin |  | 1 | 6 |  |  |  |  |
| Norfloxacin |  | 3 | 2 | 1 |  |  |  |
| Ofloxacin |  | 2 | 1 | 1 | 1 |  |  |
| Penicillin | 2 |  |  |  |  |  |  |
| Piperacillin/Tazobactum | 4 | 13 | 11 | 4 | 3 | 1 | 1 |
| Teicoplanin | 1 |  |  |  |  |  |  |
| Tetracycline | 6 | 5 | 1 | 1 | 1 |  |  |
| Tobramycin |  | 1 |  |  | 1 |  |  |
| Vancomycin | 23 |  |  |  |  |  |  |

*The number in each box indicates the number of isolates of an organism that are sensitive to an antimicrobial agent. The red colour indicates more isolates are sensitive and blue colour indicates lower number of isolates showing sensitivity for a given micro-organism*

**Table 4**: **Antimicrobial resistance pattern of the SSI isolates**

|  | Gram  Positive | Gram  Negative | | | | | |
| --- | --- | --- | --- | --- | --- | --- | --- |
|  | *Staphylococcus (n=23)* | *Klebsiella (n=13)* | E.Coli (n=12) | *Citrobacter (n=4)* | *Acinatobacter (n=3)* | *Proteus mirabilis (n=1)* | *Providencia (n=1)* |
| Amikacin |  |  |  | 1 |  |  |  |
| Amoxycillin |  | 2 | 1 | 3 | 1 |  |  |
| Amoxyclav | 5 | 10 | 2 | 3 | 2 | 1 |  |
| Amikacin |  | 5 | 3 | 2 | 1 | 1 |  |
| Ampicillin | 2 | 11 | 2 | 2 |  | 1 | 1 |
| Ampicillin/Sulbactum | 1 |  |  |  |  |  |  |
| Azithromycin | 7 |  |  |  |  |  |  |
| Aztreonam | 1 | 3 | 3 | 4 |  |  |  |
| Cefepime |  | 4 | 3 | 3 |  | 1 |  |
| Cefixime |  | 1 |  |  |  |  |  |
| Cefoprerazone/Sulbactum |  |  | 1 | 1 |  |  |  |
| Cefotaxime |  | 5 |  | 2 |  | 1 |  |
| Cefoxitin | 4 | 1 | 3 | 3 | 2 |  |  |
| Ceftazidime/Clavilanic acid | 1 |  | 2 | 2 |  |  |  |
| Ceftriaxone |  |  | 3 | 3 |  |  |  |
| Cefuroxime | 5 |  |  |  |  |  |  |
| Ciprofloxacin | 1 | 2 | 2 |  |  |  |  |
| Clindamycin | 4 |  |  |  |  |  |  |
| Co-Trimoxazole | 10 | 3 | 1 | 1 |  | 1 |  |
| Doxycycline |  | 2 |  |  |  |  |  |
| Erythromysin | 1 |  |  |  |  |  |  |
| Gentamycin | 1 |  |  |  |  |  |  |
| Levofloxacin | 3 |  |  |  |  |  |  |
| Linezolid | 1 |  |  |  |  |  |  |
| Nitrofurantoin |  | 2 |  | 1 |  |  |  |
| Penicillin | 17 |  |  |  |  |  |  |
| Piperacillin/Tazobactaum |  |  | 1 | 1 |  |  |  |
| Tetracycline | 1 | 3 |  |  |  | 1 |  |

*The number in each box indicates the number of isolates of an organism that are resistant to an antimicrobial agent. The red colour indicates more isolates are resistant and blue colour indicates low resistance for a given micro-organism*

**Discussion**

In this large prospective study, the incidence of SSI was 4.6% with a single dose prophylactic antibiotic as per the WHO guidelines, detected within 30 days post-operative period as per CDC criteria. The incidence of SSI is low compared to most Indian studies, which have reported a burden ranging from 3.12% to as high as 24.2% over the last decade. [7,14–16,19–21] Most of these studies did not follow the WHO guidelines on pre-operative antibiotic use, and some even used multiple antibiotics in multiple doses before or after surgery. [15,19–21] Therefore, we recommend adherence to the guidelines unless there is evidence-based justification to deviate from them.

We found that obesity is a strong risk for developing SSI. A meta-analysis has revealed that obese pregnant women have a higher risk of wound infections in all settings. [22] To prevent SSI in obese women, various guidelines recommend higher dose of antibiotics based on several studies. [23–25] However, these guidelines were advocated after commencement of our study. Based on these facts, we have changed our institutional policy to include double dose of antibiotic prophylaxis for obese women. We expect that this practice will further reduce the SSI burden in our setting.

Our study found that the younger (Aged <25 years) are at high risk of developing SSI after CS. In India, inconsistent results are found in the literature in this regard. [10,14,20,26] We found higher risk with emergency CS with borderline significance; but most studies in literature have confirmed a higher risk of SSI with emergency CS. [16,27,28]

In the present study, Staphylococcus aureus, a gram-positive organism, was the commonest organism isolated from caesarean wound infections. The other common organisms that were isolated include Klebsiella and E.coli, both being gram-negative organisms. The finding is consistent with the findings from other Indian studies which has reported mixed infections with gram negative and gram-positive organisms. [14–16,20]

The strengths of the present study were that it was done prospectively, single dose antibiotic was prescribed as per the WHO guidelines, and the women were followed for 30 days post-operation as per the CDC criteria. Another strength of this study is that the proforma was filled by study nurses only, to rule out reporting bias. However, the study was based on a single centre, and thus, the results might not be generalizable to the other contexts.

**Conclusion**

Since the SSI rate following single-dose antibiotic prophylaxis is 4.6% with 99% being superficial and obesity is a risk factor, it is rational to practice National and International recommendations for antibiotic prophylaxis for all women including obese, undergoing Cesarean section delivery. Our finding also indicates the need for continuous vigilance on SSI control measures at the hospital-level.

**References**

1.	World Health Organization. WHO Statement on Caesarean Section Rates [Internet]. 2015 [cited 2023 Jan 2]. Available from: https://apps.who.int/iris/bitstream/handle/10665/161442/WHO_RHR_15.02_eng.pdf

2.	Boerma T, Ronsmans C, Melesse DY, Barros AJD, Barros FC, Juan L, et al. Global epidemiology of use of and disparities in caesarean sections. The Lancet. 2018 Oct 13;392(10155):1341–8.

3.	Lancet T. Stemming the global caesarean section epidemic. The Lancet. 2018 Oct 13;392(10155):1279.

4.	Roy N, Mishra PK, Mishra VK, Chattu VK, Varandani S, Batham SK. Changing scenario of C-section delivery in India: Understanding the maternal health concern and its associated predictors. J Family Med Prim Care. 2021 Nov;10(11):4182–8.

5.	Larsson C, Djuvfelt E, Lindam A, Tunón K, Nordin P. Surgical complications after caesarean section: A population-based cohort study. PLoS One. 2021 Oct 5;16(10):e0258222.

6.	Liu S, Liston RM, Joseph KS, Heaman M, Sauve R, Kramer MS. Maternal mortality and severe morbidity associated with low-risk planned cesarean delivery versus planned vaginal delivery at term. CMAJ. 2007 Feb 13;176(4):455–60.

7.	Hirani S, Trivedi NA, Chauhan J, Chauhan Y. A study of clinical and economic burden of surgical site infection in patients undergoing caesarian section at a tertiary care teaching hospital in India. PLOS ONE. 2022 Jun 3;17(6):e0269530.

8.	World Health Organization. WHO recommendations for prevention and treatment of maternal peripartum infections. World Health Organization; 2015.

9.	Shree R, Park S, Beigi R, Dunn S, Krans E. Surgical Site Infection following Cesarean Delivery: Patient, Provider, and Procedure-Specific Risk Factors. Amer J Perinatol. 2015 Sep 7;33(02):157–64.

10.	Saeed KB, Corcoran P, Greene RA. Incisional surgical site infection following cesarean section: A national retrospective cohort study. Eur J Obstet Gynecol Reprod Biol. 2019 Sep;240:256–60.

11.	Zejnullahu VA, Isjanovska R, Sejfija Z, Zejnullahu VA. Surgical site infections after cesarean sections at the University Clinical Center of Kosovo: rates, microbiological profile and risk factors. BMC Infect Dis. 2019 Aug 28;19(1):752.

12.	Kvalvik SA, Rasmussen S, Thornhill HF, Baghestan E. Risk factors for surgical site infection following cesarean delivery: A hospital-based case–control study. Acta Obstetricia et Gynecologica Scandinavica. 2021;100(12):2167–75.

13.	Ferraro F, Piselli P, Pittalis S, Ruscitti LE, Cimaglia C, Ippolito G, et al. Surgical site infection after caesarean section: space for post-discharge surveillance improvements and reliable comparisons. New Microbiol. 2016 Apr;39(2):134–8.

14.	De D, Saxena S, Mehta G, Yadav R, Dutta R. Risk Factor Analysis and Microbial Etiology of Surgical Site Infections following Lower Segment Caesarean Section. International Journal of Antibiotics. 2013 Sep 1;2013:e283025.

15.	Gupta S, Manchanda V, Sachdev P, Kumar Saini R, Joy M. Study of incidence and risk factors of surgical site infections in lower segment caesarean section cases of tertiary care hospital of north India. Indian J Med Microbiol. 2021 Jan;39(1):1–5.

16.	Panwar D, Jodha BS, Prakash P. Study of surgical site infection: An obstetrical surgical morbidity at a tertiary level hospital | Panwar | Clinical Surgery Research Communications. 2021 Sep 29 [cited 2023 Jan 2]; Available from: http://www.antpublisher.com/index.php/CSRC/article/view/391

17.	Mangram AJ, Horan TC, Pearson ML, Silver LC, Jarvis WR. Guideline for prevention of surgical site infection, 1999. Hospital Infection Control Practices Advisory Committee. Infect Control Hosp Epidemiol. 1999 Apr;20(4):250–78; quiz 279–80.

18.	Misra A. Ethnic-Specific Criteria for Classification of Body Mass Index: A Perspective for Asian Indians and American Diabetes Association Position Statement. Diabetes Technol Ther. 2015 Sep 1;17(9):667–71.

19.	Vijayan CP, Mohandas S, Nath AG. Surgical Site Infection Following Cesarean Section in a Teaching Hospital. INTERNATIONAL JOURNAL OF SCIENTIFIC STUDY. 2016;3(12):97–101.

20.	Thakur N, Kujur A. Microbiological and antibiotic sensitivity pattern of surgical site infection following caesarean section in a tertiary care center of Chhattisgarh. International Journal of Reproduction, Contraception, Obstetrics and Gynecology. 2021 Jun 28;10(7):2638–46.

21.	Dahiya P, Gupta V, Pundir S, Chawla D. Study of Incidence and Risk Factors for Surgical Site Infection after Cesarean Section at First Referral Unit. 2016;3(4).

22.	Słabuszewska-Jóźwiak A, Szymański JK, Jóźwiak Ł, Sarecka-Hujar B. A Systematic Review and Meta-Analysis of Wound Complications after a Caesarean Section in Obese Women. J Clin Med. 2021 Feb 10;10(4):675.

23.	Coleman J, Murtha A, Silverman NS. ACOG Practice Bulletin No. 199: Use of Prophylactic Antibiotics in Labor and Delivery. Obstetrics & Gynecology. 2018 Sep;132(3):e103.

24.	Malhotra J, Mittal P, Rajaram S, Bharti R. FOGSI Focus Surgical Skills [Internet]. First. 2018 [cited 2023 Mar 9]. Available from: https://www.fogsi.org/wp-content/uploads/fogsi-focus/FOGSI-Focus-Surgical-Skills.pdf

25.	World Health Organization. WHO recommendation on prophylactic antibiotics for women undergoing caesarean section [Internet]. 2021 [cited 2023 Mar 9]. Available from: https://apps.who.int/iris/bitstream/handle/10665/341865/9789240028012-eng.pdf

26.	Sangwan A, Malhotra V. Assessment of risk factors for surgical site infection following caesarean section. International Journal of Reproduction, Contraception, Obstetrics and Gynecology. 2019 Oct 23;8(11):4518–24.

27.	Chhetry M, Subedi S, Banerjee B. Risk factors for post caesarean surgical site infection at a tertiary care center in Eastern Nepal. J Coll Med Sci-Nepal. 2017 Oct 19;13(3):314–7.

28.	Shrestha S, Shrestha R, Shrestha B, Dongol A. Incidence and risk factors of surgical site infection following cesarean section at Dhulikhel Hospital. Kathmandu Univ Med J (KUMJ). 2014;12(46):113–6.

**Acknowledgements**:

We are very grateful to our Study Nurses Ms Harati, Mrs Rosamma, study Co-ordinators Mrs. Deepa and Mrs Mamta and our IT team Mr. Purushotham Reddy and Mr. Narender.
