# Supplementary material for: Prospective cohort study of surgical site infections following single dose antibiotic prophylaxis in caesarean section at a tertiary care teaching hospital in Medchal, India
Source: PLoS One. 2024 Jan 25;19(1):e0286165. doi: 10.1371/journal.pone.0286165 (PMC10810521; doi:10.1371/journal.pone.0286165)
Supplement: Attachment — Submitted filename: Response to Reviewers.docx [file pone.0286165.s004.docx]

**Response to Reviewers**

Page 2, Line 34: “………….profiles and the antimicrobial sensitivity and resistance pattern………..”

Comment: Replace ‘antimicrobial sensitivity and resistance pattern’ with ‘antimicrobial susceptibility’ only. Please use the word ‘susceptibility’ instead of ‘sensitivity’ throughout the manuscript.

Response: Agree with the suggestion, replaced with the word ‘ susceptibility’.

Page 2, Line 49: “Of these, 92 participants……..”

Comment: Delete ‘Of these’ and start the sentence with ’Ninety-two participants’.

Response: Agree with the suggestion , changed.

Page 2-3, Lines 54-55: “Microbial growth was observed in 75.8% 3 55 (n=50/66) samples”

Comment: Please rephrase the above sentence like “Microbial growth in culture was observed from 55 (75.8%) out of total 66 samples”. Please mention the statistical figures in and out of the parentheses within a sentence uniformly throughout the manuscript.

Response: As suggested by the reviewers,the sentence was rephrased. The statistical figures were mentioned uniformly throughout the manuscript.

Page 3, Lines 55-56: The most common organisms identified were Staphylococcus aureus 56 (n=23, 46.0%), Klebsiella sp. (n=13, 26.0%), and Escherichia coli (n=12, 24.0%).

Comment: From the above sentence, it appears to be that the percentages of the organisms were calculated considering the total number of samples with a positive growth as denominator. Since multiple organisms have been isolated from 7 samples (as mentioned under Results), it will be helpful if the percentages are calculated considering total number of isolates in the denominator. Please also make relevant changes in the text under ‘Results’ section

Response: Thanks for the suggestion. We have done the calculation accordingly. Also, as suggested by the other reviewer, we have also reported the number of Staphylococcus aureus isolates and number of Coagulase negative Staphylococcus isolates.

Page 3, Lines 58-61: Conclusion: Given the low rate of SSI following Caesarean deliveries subjected to single-dose antibiotic prophylaxis and the increased risk noted with obesity, it is rationale to practice the latest recommendations of WHO including higher dose for obese patients, unless there is compelling evidence to do otherwise in any context.

Comment: It is strongly advisable that the author should rewrite the conclusion of both Abstract and the main text based on the objective of the study only i.e. rate of CS and significant risk factors. Any recommendation based on the findings, may be included under the Discussion only.

Response: As suggested, conclusion was changed.

Page 4, Lines 83-84: To prevent SSI, the WHO recommend using a single dose antibiotic, mostly the first generation cephalosporins, before 30 to minutes of incision for all women undergoing CS.

Comment: As per the reference cited, the recommendation for as single dose antibiotics is first generation cephalosporins or penicillin. Authors are advised to recheck the same and make required changes

Response: Rechecked the reference no.8. Changes made

Page 5, Lines 107: We classified CS into two types- emergency and elective.

Comment: In general, emergency procedures have more risk for post-operative infections than elective procedures. As more than 60% of the patients underwent emergency CS it will be better if some comparative analysis is done based on the parameters described in the manuscript between emergency and elective CS

Response: As advised by the reviewers, comparative analysis was done between elective and emergency CS

Page 5, Line 113: For estimating 2% or less incidence of SSI after CS

Comment: Please clarify on the incidence of SSI after CS for calculating sample size. Or cite suitable reference.

Response: We thank the reviewer for the comment. According to the study conducted by Vijayan et al (2016), we determined that a minimum sample size of approximately 1500 women undergoing caesarean section would be necessary to estimate a 4.1% incidence of surgical site infections (SSI). This estimation took into account a significance level (α) of 0.05, a power (β) of 0.8, and a margin of error of 0.01.

Reference: Vijayan CP, Mohandas S, Nath AG, Surgical site infection following caesarean section in a teaching hospital. International Journal of Scientific Study. 2016;3(12);97-101.

Page 5, Lines 116-119: When Cefazolin was unavailable, injection Ampicillin 1 gram, OR injection Cefotaxim 1 gram was given.

Comment: Check the spelling of antibiotics. Few are written wrongly in the manuscript. Within a sentence, the name of the antibiotics should be written in lower case letters only.

As per the above-mentioned sentence, a third generation of cephalosporin was sometimes chosen if cefazolin, a first-generation cephalosporin, was not available. Is this also a part of hospital’s antibiotic policy?

Also mention under discussion why choosing a higher generation cephalosporins instead of first generation cephalosporins only will not cause any difference in rate of SSI in the study.

Response: Spelling of cefotaxime changed. Yes, it is the hospital policy to use third generation cephalosporin if first generation of cephalosporin was not available.

Page 6, Lines 121-124: Definition of variables: SSI was defined as- "An infection of the superficial or deep skin incision, or of an organ or space, occurring up to 30 days after surgery.” [17] There are two types of SSI- superficial and deep. As per the CDC definition, "A superficial SSI is infection identified during hospital stay or within 30 days……..

Comment: It is not clear what exact criteria were adopted for defining SSI. From the later sentences of the above paragraph, it seems to be that the authors have adopted CDC surveillance definitions. If NHSN-CDC surveillance definitions were considered to define the cases in the study, that should be directly cited as reference. Moreover, considering the detailed nature of the definitions of the guidelines it should be enough to state that “SSI for the present study was defined as per the CDC-NHSN guidelines”.

Response: As suggested , definition of SSI was changed accordingly, reference given

Page 7, Lines 150-151:  Data collection: All pertinent clinical details were collected by from the participants hospital record using structured questionnaire by a dedicated study nurse.

Comment: Please upload the structured questionnaire as supplementary file. Please also mention about the source of the questionnaire under the ‘Methods’ section.

Response: The questionnaire was uploaded as supplementary file.

Page 7, Lines 152-171: The hospital policy was to discharge the patient after suture removal………………….. microbiological procedure was followed for those who were discharged and followed up in the OPD.

Comment: Please provide some headings for the above paragraphs like “Laboratory diagnosis of SSI”, “Post-discharge Follow up”.

Response: As advised by the reviewers, headings were provided.

Page 10, Lines 200-202: The average hospital stay was 11.8 days (SD 3.7 days) in women who developed SSI and 9.3 (SD 3.1) days without SSI and mean difference was 2.5 days (95% CI: 1.9 to 3.2 days; p <0.001).

Comment: Please indicate under Statistical Methods in the Methods section about the specific test that was used for comparing the continuous quantitative variables.

Response: We agree with the reviewers. As suggested, mentioned in the statistical methods

Page 11, Lines 215-216: The only gram-positive organism, Staphylococcus, was commonly sensitive to- Linezolid (20 isolates, 87%)….

Comment: Authors need to recheck and confirm the susceptibility profile of staphylococcal isolates against linezolid. Linezolid resistance in Staphylococcus is still very low in India. No speciation has also been done for Staphylococcus. Please differentiate between Staphylococcus aureus and coagulase-negative staphylococcus in the manuscript.

Response: We thank the author for the comment. We have discussed the same with the clinical Microbiologist and reanalyzed the figures. There were 7 isolates of Staphylococcus aureus and we have reported the sensitivity and resistance based on these 7 isolates. We have differentiated between the two organisms, viz. Staphylococcus aureus and coagulase-negative staphylococcus.

Page 12, Lines 221-222: ……….. and Cefepime (9 isolates, 69.2%) and commonly resistant to Ampicillin (11 isolates, 222 84.6%) and Amoxycillin/Clavulanic acid combination (10 isolates, 76.9%).

Comment: It seems to be that the intrinsic resistance was not considered for some of the organisms in the study. Those with intrinsic resistance against some particular organism, need not to be analysed for resistance/susceptibility profile against that particular organisms. Authors are strongly encouraged to seek help from clinical microbiologists or ID specialists for these technical issues

Response: We have discussed with the Clinical Microbiologist and considered the intrinsic resistance and removed those antibiotics. We have merged table 3 and table 4 as suggested by the other reviewers also.

Page 16, Lines 246-247: Therefore, we recommend adherence to the guidelines unless there is evidence-based justification to deviate from them.

Comment: The statement may be overly correct, as the authors themselves did not uniformly followed the WHO guidelines (choice of antibiotics) for all the subjects.

Response: Yes. We understand that and we have reported that practicality to avoid any biased statement.

Page 17, Lines 262-263: The finding is consistent with the findings from other Indian studies which has reported mixed infections with gram negative and gram-positive organisms. [14–16,20]

Comment: The authors should rephrase the above sentence as mixed infections term may be misleading and often refers to co-infections by both types of organisms mentioned above

Response: The sentence has been rephrased.

Page 17, Lines 266-267: Another strength of this study is that the proforma was filled by study nurses only, to rule out reporting bias.

Comment: Authors should also mention about the questionnaire filled up by the nurses under the ‘Methods’ section

Response: As suggested by the reviewers , mentioned under the ‘Methods’ section
