# Supplementary material for: Prospective cohort study of surgical site infections following single dose antibiotic prophylaxis in caesarean section at a tertiary care teaching hospital in Medchal, India
Source: PLoS One. 2024 Jan 25;19(1):e0286165. doi: 10.1371/journal.pone.0286165 (PMC10810521; doi:10.1371/journal.pone.0286165)
Supplement: Attachment — Submitted filename: Responses to the Reviewers.docx [file pone.0286165.s005.docx]

Responses to the reviewer’s comments

**Editors comment:**

1. The exponentiated coefficients for logistic regression model are interpreted as odds ratios whereas for log-binomial regression they are relative risk ratios. The authors have mentioned that they have conducted logistic regression but mentioned relative risks instead of odds ratio. This needs clarification.

Response: We thank the editor for pointing it out. We have revised the terminology used from ‘logistic regression’ to ‘binomial regression”. Vide line number 174-176.

**Editors comment:**

2. Please edit the title of the manuscript while resubmission and delete "Dear Editor".

Response: Thanks. We will ensure that while resubmitting the manuscript.

**Reviewers # 3 comments**

3. The statistical analysis is not in accordance with the research question. The research question is whether there is a reduction in the SSI following single antibiotic prophylaxis but the analysis is on various risk factors rather.

Response: We thank the reviewer for the comment. Please note that our primary objective was- to determine the incidence of post CS SSI following the adoption of single-dose antibiotic prophylaxis as recommended by WHO [8] in a tertiary care teaching hospital in Medchal, Telangana state, India. (Vide- line number 93-95).

Besides, we had two more secondary objectives- “Additionally, we looked at the risk factors of SSI and reported the bacteriological profiles and the antimicrobial susceptibility pattern of the culture positive isolates.” (Vide line number 99-101)

In our results, we have articulated the results in the same line- the incidence of SSI, the risk factors and the susceptibility pattern of the culture positive isolates.

All the authors have gone through the results and we strongly feel that the statistical analysis is in accordance with the research objectives.

4. The study variables are not well defined.

Response: We have added the definitions of all other variables those were missed out in the previous draft with appropriate references. (Vide line number 139 to 145). Please note that we have revised the sequence of references as well while incorporating the additional references.

5. The study population is not homogenous which includes both elective and emergency caesarean section group.

Response: We thank the author for the comment. Please note that in a hospital setting, we expect both elective and emergency caesarean section group. Hence, we believe that the population is homogeneous. In addition, we strongly feel that type of surgery may be directly related to SSI. Hence, we have reported the primary outcome of interest (SSI incidence) separately for both these groups to ensure clarity in the results.

6. The elective section group included by the author also has patients in prolonged labour which is incorrect.

Response: We agree with the reviewer and we have done the necessary correction in table 3.

7. There is no comparator group. The institute's previous SSI at least should be mentioned.

Response: As our primary outcome was to calculate the SSI incidence, we did not require a comparator group. Also, taking a historical control has it’s own challenges, especially the robustness of the information. Hence, we feel that taking a comparator group might not help improving the interpretation of the present finding. However, to calculate the risk, we have used the comparator group.

**Reviewers #4 comment**

8. It seems that these lines depicting resistance/susceptibility profile of Ampicillin in Klebsiella species has not been modified as suggested by other reviewers. Authors are suggested to check the intrinsic resistance profile of Klebsiella species and accordingly make changes in the manuscript.

Response: We thank the reviewer for pointing it out. Comment of the earlier reviewer was “It seems to be that the intrinsic resistance was not considered for some of the organisms in the study. Those with intrinsic resistance against some particular organism, need not to be analysed for resistance/susceptibility profile against that particular organisms. Authors are strongly encouraged to seek help from clinical microbiologists or ID specialists for these technical issues.”

Though we changed mostly based on the comment, we might have missed a few inadvertently. We have now incorporated the changes as suggested after discussing with the clinical microbiologist. The changes are as follows:

- The ampicillin resistance pattern for Klebsiella has been removed from the table and from the text. (Vide table 4, line number 227-8 in the text)
- For Staphylococcus aureus (Table 4), we have removed Methicillin as the Methicillin susceptibility/resistance was screened by cefoxitin). We have also removed Aztreonam and Vancomycin resistance/susceptibility pattern for Staphylococcus aureus. (Vide table 4, line number 223).
