# Supplementary material for: Prospective cohort study of surgical site infections following single dose antibiotic prophylaxis in caesarean section at a tertiary care teaching hospital in Medchal, India
Source: PLoS One. 2024 Jan 25;19(1):e0286165. doi: 10.1371/journal.pone.0286165 (PMC10810521; doi:10.1371/journal.pone.0286165)
Supplement: Attachment — Submitted filename: Responses to the Reviewers - Copy.docx [file pone.0286165.s006.docx]

Responses to reviewers

**Reviewers # 3 comments**

1. Labouring patients are included in the elective section group in the table

Response: We thank the reviewer for pointing it out. We have gone through the data source carefully and learned that an inadvertent error took place from our end , and we misclassified a few participants as elective section group, whereas, they should have been included in the emergency CS group. Hence, we have updated the data and reanalyzed it and updated the results and the discussion section as per the need. We have also replaced the previous data that we submitted to PLOS One with the updated data.

2. Grammatical and punctuation errors needs to be corrected

Response: Thank you the reviewer for pointing out. We have addressed them.

3. The names of the antibiotics like methicillin, ceftazidime, etc. and terms like caesarean delivery, elective need not be capitalised

Response: As suggested, we have changed into small letters.

.

**Reviewers #4 comment**

Authors have mentioned that all the isolates of Staphylococcus were sensitive to Methicillin (Table 4). (line number 223), but have not shown the same in table 4. As per CLSI guidelines, though Methicillin susceptibility/resistance was screened by cefoxitin, it should be mentioned in the table and the isolate should be reported in results as Methicillin sensitive Staphylococcus aureus (MSSA)

Response: We thank the reviewer for the comment. We have made necessary changes in the table.
