# Supplementary material for: Prospective cohort study of surgical site infections following single dose antibiotic prophylaxis in caesarean section at a tertiary care teaching hospital in Medchal, India
Source: PLoS One. 2024 Jan 25;19(1):e0286165. doi: 10.1371/journal.pone.0286165 (PMC10810521; doi:10.1371/journal.pone.0286165)
Supplement: Attachment — Submitted filename: Responses to reviewers.docx [file pone.0286165.s007.docx]

Responses to Editor comments

1. Authors have made few changes in the statistical figures but similar changes have not been made in the Abstract

Response: Thank the Editor for pointing out. Statistical figures have been changed.

2. As per Table 1, the total number of patients for whom data on labour was available are 2015. But the same has been mentioned as 2010 in Table 3.

Response: As suggested, the changes have been made.

3. In Table 3, the percentage of participants in the Emergency CS group with <6 hours duration of labour has been wrongly mentioned

Response: Thank the Editor for pointing out. We corrected the percentage.

4. In Table 3, the number of participants in the Elective CS group (non-labour) should be 783 not 78.3

Response: In Table 3, the number of participants in the Elective CS group was mentioned as 783.
